# The perceptions of danish physiotherapists on the ethical issues related to the physiotherapist-patient relationship during the first session: a phenomenological approach

**DOI:** 10.1186/1472-6939-12-21

**Published:** 2011-10-12

**Authors:** Jeanette Praestegaard, Gunvor Gard

**Affiliations:** 1 Dept of Physiotherapy Health Sciences, Health Sciences Center, Lund University, Box 157, 221 00 Lund, Sweden; 2 Dept of Physiotherapy, Metropolitan University College, Sigurdsgade 26, 2200 Copenhagen, Denmark; 3 Department of Health Sciences, Luleå University of Technology, 971 87 Luleå, Sweden

## Abstract

**Background:**

In the course of the last four decades, the profession of physiotherapy has progressively expanded its scope of responsibility and its focus on professional autonomy and evidence-based clinical practice. To preserve professional autonomy, it is crucial for the physiotherapy profession to meet society's expectations and demands of professional competence as well as ethical competence. Since it is becoming increasingly popular to choose a carrier in private practice in Denmark this context constitutes the frame of this study. Physiotherapy in private practice involves mainly a meeting between two partners: the physiotherapist and the patient. In the meeting, power asymmetry between the two partners is a condition that the physiotherapist has to handle. The aim of this study was to explore whether ethical issues rise during the first physiotherapy session discussed from the perspective of the physiotherapists in private practice.

**Methods:**

A qualitative approach was chosen and semi-structured interviews with 21 physiotherapists were carried out twice and analysed by using a phenomenological framework.

**Results:**

Four descriptive themes emerged: general reflections on ethics in physiotherapy; the importance of the first physiotherapy session; the influence of the clinical environment on the first session and; reflections and actions upon beneficence towards the patient within the first session. The results show that the first session and the clinical context in private practice are essential from an ethical perspective.

**Conclusions:**

Ethical issues do occur within the first session, the consciousness about ethical issues differs in Danish physiotherapy private practice, and reflections and acts are to a lesser extent based on awareness of ethical theories, principles and ethical guidelines. Beneficence towards the patient is a fundamental aspect of the physiotherapists' understanding of the first session. However, if the physiotherapist lacks a deeper ethical awareness, the physiotherapist may reason and/or act ethically to a varying extent: only an ethically conscious physiotherapist will know when he or she reflects and acts ethically. Further exploration of ethical issues in private practice is recommendable, and as management policy is deeply embedded within the Danish public sector there are reasons to explore public contexts of physiotherapy as well.

## Background

Physiotherapy in private practice involves mainly a meeting between two partners: the physiotherapist and the patient. In the meeting, power asymmetry between the two partners is a condition that the physiotherapist has to handle. The asymmetry of power is based on the partner's power inequality in defining and setting the situation: generally, the one needs help and the other has the adequate skills to offer assistance [1]. In addition to laws and guidelines as regulation of the professional interaction, it is the physiotherapist who is in power to define the setting, ask questions, set the timeframe e.g. and thereby has the professional responsibility to ensure the patient equal status within the session.

In addition to power asymmetry, physiotherapy is characterised by a close and often continued relationship between physiotherapist and patient, both by being touched by one another physically and mentally [2,3] and by the fact that the responsibility for success or failure to a great extent is shared between the physiotherapist and the patient [2].

Thornquist writes that every meeting between physiotherapist and patient implies both written and unwritten codes of conduct. And even when meetings are planned and roles and codes of conduct seem fairly clear-cut it is never given how they shall go. Something is given and something is brought about between the partners [1].

Communication within the professional meeting has been reduced to a linear transfer of information [4] where interpretation and context were neglected [1]. Our point of departure is Thornquist's perspective on communication as a fundamental social activity; a mutual exchange of meaning, a dialogue where we address each other to create reciprocity in experience and meaning. Focus is on what happens between the partners both verbally and non-verbally, on the basis that both partners have their own understandings and interpretations of values, norms and traditions [1]. Additionally, we see the organisational and institutional structures and the clinical environment where the two partners meet as having influence on what happens. In communication we exchange messages. Messages always come out from a context, and they are always interpreted in a context, but this may not be the same context as they come out from. What the partners do or don't do, say or don't say, are all actions that set the context.

### The characteristics of the meeting in physiotherapy

A meeting in physiotherapy private practice is set in an organisational but also in a meaningful frame [1]. Both are essential to unfold to set the context of the aim of the study.

Today, almost 40% of all Danish physiotherapists are employed in private practice. The rest are employed within the public setting; in hospitals, in municipal institutions, in university colleges etc. Increasingly, students attend clinical courses in private clinics, and it is becoming more and more popular to choose a carrier in private practice rather than in public employment, typically motivated by the feeling of professional freedom and by the opportunity to make a solid profit [5,6]. By Danish law, physiotherapy in private practice is granted federal subsidies, so people receiving physiotherapy pay half the cost and the government covers the rest. A typical clinic in Denmark is owned by one, often senior, physiotherapist, who has two - four physiotherapists leasing in at the clinic.

Persons who attend private physiotherapy have all sorts of acute problems, e.g. sports injuries', a crick in one's neck or back, a tennis elbow or sub-acute problems as low back pain, muscle tensions etc. People with severe physical handicaps or functional limitations due to progressive sickness, may through medical assignment be granted cost-free physiotherapy in private practice. Furthermore, some private clinics offer home treatments to patients who are too ill to get to the clinic, or palliative patients. Within this complex professional setting, ethical competences are required to handle all aspects of actions in clinical practice.

We use the term *Ethics *in reference to Purtilo, who states: "Ethics is a systematic reflection on morality: *Systematic *because it is a discipline that uses specific methods and approaches to examine moral situations and *reflection *because it consciously calls into question assumptions about existing components of moralities that fall into the category of habits, customs, or traditions." [7]

We emanate from the understanding that ethical issues are relational situations where one needs to weigh alternative actions towards a moral problem [8] and that ethical issues are embedded in every clinical meeting [2,3,5,7]. Ethical issues in relation to physiotherapy private practice can be about how to manage the power asymmetry to ensure both parties feel humanly equal, how to communicate in a respectful manner with all clients, how to live up to the right of self-determination and privacy, or how to deliver client-orientated therapy to all kinds of patients [5,9-11].

### Knowledge about ethical dimension of physiotherapy in private practice

Despite the increased political and professional recognition of the ethical dimension in physiotherapy, a limited amount of articles has been published on the topic. In 2002, Swisher reviewed the knowledge on ethics present in physiotherapy literature from 1970 - 2000 [12]. Swisher concluded that very few studies, actually only 5 publications [13-17] attempted to define ethical issues' which physiotherapists routinely face in practice. Cross and Sim in 2000 showed that physiotherapists may be lacking in awareness about policies and procedures relating to confidentiality, which could lead to undesirable consequences for clinical practices [18]. In 2001, Praestegaard concluded from an interview study of 17 physiotherapists that Danish physiotherapists were interested in the ethical dimension of physiotherapy but not consciously aware of when, why or how often ethical issues occurred in practice. This low degree of awareness led to several immoral and sometimes even illegal actions, particular in the context of private practice [5]. Carpenter et al concluded in 2008 that the impact of institutional environment on generating ethical issues and on practitioners' management of them needed more systematic investigation [19].

Only sparse research has focused on ethical issues within the context of private practice. Potter et al [9-11] published three studies about physiotherapy in private practice and discovered several ethical issues within this context. They found that both physiotherapist and patient expected the other to show respect and trust, to be honest, caring and empathetic [9], that patients' negative experiences of physiotherapy related to poor communication skills [10], and that communication skills and behaviour modification techniques were strategies physiotherapists wanted to learn more about when dealing with difficult patients in order to provide ethically physiotherapy in private practice [11]. In 2006, Greenfield pointed out the difficulties in applying an 'ethics of care' approach to practice in health management contexts, where a focus on cost-effectiveness and profit was more common [20]. And, in 2007, Delaney published an interview study of the interpretation of informed consent in private practice. She found that physiotherapists defined informed consent as an implicit component of their routine clinical presentation and information, rather than a process of providing explicit patient choices [21]. No studies have been found on ethical issues relating to first sessions in physiotherapy private practice. The aim of this study has been to explore whether ethical issues arise during the first physiotherapy session discussed from the perspective of the physiotherapists in private practice.

## Methods

### Study design

This study was designed within a qualitative paradigm, using a phenomenological approach [22], in particular Malterud's modification of Giorgi's phenomenological analysis [23-25].

Initially, the aim of this study was to explore whether ethical issues arise during the process of physiotherapy discussed from the perspective of the physiotherapists in private practice. Early in the interview process, it became clear that the interviewees found it important to distinguish between the first session and the subsequent physiotherapy sessions. They found the first session to be something special to be ethically aware about and, therefore, this study and its interview guide were adjusted towards reflections and actions within the first session.

Two interviews were performed as earlier research show that physiotherapists in Denmark generally have vague ethical awareness [5]. By giving time to reflect upon the subject of the study, we assumed the interviewees would provide deeper and more reflected answers, so the second interview was also a triangulation tool [25]. A semi-structured interview guide was developed and included open-ended questions. In the first interview, the questions focused on: reflections and narratives on the first session of physiotherapy in private practice, reflections about the constitution of an optimal first session, and discussions of ethical issues emerging within the first session. The second interview focused on: reflections and/or adjustments on the first interview, further reflections and narratives about optimal and/or regrettable meetings and professional conduct related to the first sessions.

### Sampling

Our sampling strategy aimed at obtaining a sample of physiotherapists in private practice with a wide range of experiences due to our assumption that ethical issues can emerge in any clinical meeting.

### Procedures

An invitation letter introducing the subject of the study and asking for interested participants was sent out to 31 clinics across all regions in Denmark. Thereafter, the clinics were contacted by telephone and asked if they wanted to participate. Nine clinics found the study important but lacked time for participation. The rest of the clinics had passed the letter around and several physiotherapists showed interest in participating.

The selected physiotherapists should: speak fluent Danish and work in private practice irrespectively of gender, age, work position, experience or geographical region. Twenty two participants willingly agreed to participate in two interviews related to professional issues, all of which signed a written informed consent. One of the 22 was excluded due to upcoming maternity leave. For the characteristics of the interviewees see table 1.

**Table 1:** Characteristics of participants (N = 21)

Characteristics	No. (%)
**Sex**	
• Female	12 (57)
• Male	9 (43)
**Years of service in private practice**	
• 1-5	3 (14)
• 5-10	6 (29)
• 10-15	4 (19)
• 15-20	4 (19)
• 20-25	3 (14)
• 25-30	1 (5)
**Type of conditions of service**	
• Owner	11 (52)
• Renter	6 (29)
• Employed	2 (9,5)
• Self-employed	2 (9,5)
**Working individually or working together with in a clinic**	
• Individual	3 (14)
• Working in a clinic	18 (86)
**Having post graduate education**	16 (76)
**Having post graduate academic education**	5 (24)

Face-to-face, semi-structured, tape recorded interviews were conducted twice with each participant by the first author. Emerging themes and ethical issues from the first interviews were explored in the second interviews in order to stimulate the participants' ethical awareness and hereby to stimulate thickened descriptions for reflected and deeper understandings. At the second interview, it was possible further to explore, verify, refine, and add reflections and acts to earlier descriptions. The majority of the interviews were carried out in the clinic which gave a solid frame of reference for understanding and validating the comprehension of the interviewees' life world and example. A few were carried out in private homes or in a neutral office due to the participant's preference. The first interview lasted from 45 - 60 minutes, and the second from 30 - 45 minutes. The time between the two interviews varied from one to two months to allow time for settling the understanding of ethical issues within the initial encounter. One interview took five months between the two interviews due to the interviewee's business. Notes about the interview situation, the process and other impressions were written down immediately after both interviews with each participant and were used to contextualise the accounts and to help with orientation and understanding during the analysis.

Audiotapes of the interviews were transcribed verbatim by a secretary and were validated by letting the first author read the transcripts while re-listening to the interviews to ensure validity.

### Ethical considerations

The study followed the principles of the Helsinki declaration and all participants were informed about the purpose of the study and that they could stop the interview at any point without any explanation. Verbal and written informed consent and an agreement that quotes from the interviews could be used anonymously were obtained for both interviews. Approval by The Danish Research Ethics Committee is not legally required for this kind of study [26].

### Analysis

All interviews were analysed according to the principle of Giorgi's phenomenological analysis [27,28], modified by Malterud [24,25]. The analysis followed four steps:

1. Reading all transcripts to get a general sense of the whole statement. In this process, material relating to the themes about the first session was identified and put aside for later use.

2. Re-reading of the material to discriminate units with meaning from an ethical perspective.

3. Abstracting the content of meaningful units within each theme (see table 2), which resulted in four themes and appertaining subgroups. These were validated for each individual and across all interviews in discussion with the second author.

**Table 2:** Results of the analysis: themes and their appertaining subgroups

Theme	Subgroup
**General reflections on ethics in physiotherapy**	
**The importance of the first**	
**physiotherapy session**	
**The influence of the clinical environment on**	
**the first session**	
**Reflections and actions upon beneficence**	Prerequisites for the first meeting
**towards the patient within the first session**	
	Information and communication as beneficial tools
	The need for time to individualise the first session
	The influence of culture, tradition and language
	Some patients do not fit private practice
	Taking informed consent

4. Synthesising of the transformed themes into a consistent statement regarding the subject's experience. From the themes quotes were selected in order to document and root the descriptions. The quotes were translated from Danish to English by the first author and then retranslated and discussed with an external translator to maximise agreement on the content of meaning. Finally the themes were given a conclusive headline.

## Results

The results section presents issues which the interviewees consider as ethical issues. The results of the analysis emerged as four themes: General reflections on ethics in physiotherapy; the importance of the first physiotherapy session; the influence of the clinical environment on the first session and; reflections and actions upon beneficence towards the patient within the first session. The content of meaning of the themes and their appertaining subgroups are presented and illustrated by quotes in italics in the following.

### General reflections on ethics in physiotherapy

All interviewees considered ethics an important aspect of physiotherapeutic professionalism although most had difficulty explicating their understanding of ethics. Many lacked words and related to personal understandings or bodily sensations: *When I do something ethically wrong I feel it in my stomach as something unpleasant*. It seemed as the interviewees with post graduate academic education had a greater tendency to express awareness of the ethical dimension and they argued with common moral norms: *an ethical issue is an issue where you have to choose between different actions to show respect, [..] to tell the truth, to inform truthfully about diagnosis and prognosis, [..] to avoid offending the person [..] A frequent issue in private practice.*.

For me [..] an ethical issue in practice is an issue where either I don't know how to take care of the client, or, when I have to choose between two similar actions, to do so (morally) right. .. E.g. when a new client smells badly I find it an ethical issue; ought I tell the client he smells or ought I endure the smell to show my respect?

The interviewees found it important to be aware of when and how ethical issues occurred in daily practice. They expressed that they had experienced ethical issues within private practice: a few expressed that they experienced ethical issues very seldom, most experienced them daily or weekly, and a few narrated about the occurrence of several ethical issues in every meeting, as one states: *every meeting is an ethical issue because I have to treat every client differently in order to treat them with the same respect*.

### The importance of the first physiotherapy session

The interviewees considered the first session as pivotal for establishing a good physiotherapy-patient relationship for the further process of physiotherapy: *it is within the first meeting that you either win or lose your patient*. One interviewee contrasted; *every meeting is important, none more than another*.

They expressed it as essential in the first session to establish a physiotherapy-patient relationship based on trust. The majority stated that their understanding of a trustful relationship related to consciously trying to establish a respectful and empathetic dialogue, where the patient afterwards would describe the physiotherapist as being engaged and active listening to his suffering. A few were not able to describe their understanding of trust although they found it pivotal for the first session with the patient.

### The influence of the clinical environment on the first session

The majority stressed that the influence of the clinical environment on ethical reflections and actions was evident, especially on the first session. However they reflected differently from one another: most strived to arrange the clinic in order for the patient to get the impression of friendliness, trustworthiness and professionalism and they told about decorating with white walls, having designer furniture, systematic shelving units, and dressing in neutrally colored sweat suits. They described this as setting a frame of trustworthy professionalism and found it ethically sound but were not able to explain how it was ethically sound.

A few experienced interviewees explained how the clinical environment influenced the first session: *I always have the first dialogue with the patient in one of the closed rooms. The patient next door could be his worst enemy, his boss or neighbour. Of course the environment influences the quality of the physiotherapy-patient relationship*. One interviewee reflected differently: *in our clinic we have curtain as walls between the couches, and this I find very helpful because I just have to call for guidance or help and straight away my colleague is there [..] We have a very perky and flirty tone, he, he [..] both patients and each other, [..] and often I tell my colleague what I am doing and ask what to ask or do next (through the curtains)*.

The interviewees who had post graduate academic education seemed to view the concept of environment at a more abstract level. They expressed awareness that their dialogues with the patient also arose from the organisational base. They considered it an ethical dilemma because the organisational setting gave rise to limited time within the first session. In consequence of limited time, they felt they lost the possibility to gain important knowledge about the meaningfulness of the patients' lived lives. *Most of my patients expect me to examine and treat their illness, and not to ask about their lived lives. This I find as an ethical dilemma because my understanding of physiotherapy and the body is holistic and the most patients see this very narrowed; they express themselves as a machine and not as a human being*.

*I sometimes have expectations and ambitions on behalf of the patient, - that we can reach far better results. But if I don't feel or hear acceptance from the patient initially I respect that. I have to accept and respect that we have different goals. Even though I know that the patient probably can reach a higher level of activity. I must be based in the patients' needs, not my own, especially when time is scarce- otherwise it is unethical*.

### Reflections and actions upon beneficence towards the patient within the first session

The interviewees expressed great concern and effort around beneficence towards the patient and most regarded beneficence as an ethical issue to be aware about in order to respect the patients entering one's practice: *I mean, if the patient doesn't feel that I show him respect as a human being and that I am doing my best to understand and treat his problem, then I don't benefit him. Beneficence is the base of physiotherapy. It is the base of the initial meeting. Otherwise the patient may leave*.

The theme represents all types of reflections and actions intended to benefit the patient within the first session, and is presented in six subgroups: Prerequisites for the first meeting; information and communication as beneficial tools; the need for time to individualise the initial session; the influence of culture, tradition and language; some patients do not fit private practice and; taking informed consent.

#### Prerequisites for the first meeting

It seemed as some of the experienced interviewees and the interviewees with post graduate academic education, emphasised that awareness about oneself, as "who I am", was found a prerequisite for being professional within the first meeting, and the further process. They identified "who I am" as being an honest and trustworthy person: honesty about one's personal resources, values, boundaries and prejudices and as a physiotherapist: consciousness about knowledgebase and skills. *If I don't know where I end as a person and start as a physio, I cannot honestly listen to the patient. I need to be honestly aware about myself, otherwise I can lead myself and the patient to places which are not professionally good or right [..] I have the power*.

*The same sentence, question or action can be delivered without any fellow human empathy, or it can be delivered in deep respect of the person's lived life, knowledge and experiences. It is vital for an ethical first time meeting that you know where and who you are*.

The rest of the interviewees did not mention this subject.

#### Information and communication as beneficial tools

The interviewees that had post graduate academic education and the less experienced interviewees, expressed it to be ethically sound to distinguish between information and communication in order to benefit the patient: *you have to communicate with the patient, not merely inform [..] When you just inform you are being superior, and you lose the patient's active involvement in the dialogue*. They regarded consciously communication as a prerequisite for patients to develop knowledge about their problem and situation in order to develop their own strategies for problem solving and to become autonomous. Hereby they described it to be an ethical issue - especially within the first session.

The majority of the interviewees valued information as the core of beneficence towards the patient, especially within the first session. They described the first session to be like an interview: *well, in the initial meeting [..] I ask the questions and the patient answers, it is as simple as that*. They took pride in informing and educating their patients about their illness or sickness and assessed it as ethical sound physiotherapy. They wanted their patients to feel trusted and respected, and to show the patients their engagement and seriousness about their illness.

#### The need for time to individualise the first session

All interviewees agreed that lack for time in private practice in general had ethical consequences: *A lot slips out! A lot of small things fail. It is the balance between all the good intentions and the fact that we work with people that is essential. Despite all good intentions, the phone rings, a patient is having a bad day and needs more time which is not available. I am busy, too busy. I misinterpret the content. I lose control over my contact with the patient. And, [..] What can I do, the next patient is waiting*.

The need for time was expressed to be an overall ethical issue embedded within every aspect of beneficence within the first session: *The most serious ethical dilemma we have is between having time for the individual patient concurrent with making sound business*.

The majority of the interviewees stressed the difficulties in prioritising time, and they had great concerns about how to ensure a solid interview within the first session contemporary with their concerns about keeping the time schedule; the next patient arrives in 30 minutes. They argued that two patients per hour were needed in order to maintain a sound profit. They told that they tried to solve the dilemma by meeting and treating two patients at one time. Some were aware about the stress it implied for patients, and for the physiotherapist as well with the potential limitation of providing optimal physiotherapy. As one stated; *I know this is bad for both the dialogue with the one patient and the treatment effect of the other patient. But what can I do? I have to earn money. It is also a business, not just beneficial charity [..] Well of course [..] this you can probably see as an ethical issue*. Or some managed by having (too) long working hours with patients waiting up for hours. There were only sparse reflections about the impact this could have on public trust in the profession.

The interviewees with post graduate academic education, had taken action on this issue by establishing private clinics without any federal subsidies: *I have chosen to work on a 100% private basis without any kind of federal subsidies. In this way, I can fulfil my personal ethical standard; take the time I need for talking with and treating the patient the way I consider necessary in order to be doing good. I feel satisfied and the patients express respect and security*. A few scheduled 60 minutes for every new meeting to ensure a solid dialogue within the first session despite the loss of income. One interviewee with more than 25 years of service in private practice declined to talk about this issue.

#### The influence of culture, tradition and language

The female interviewees gave several examples of how culture, tradition or language rise ethical issues within the initial meeting which they found compromised being beneficent towards the patient. Some female interviewees narrated about patients who did not understand the intentions of the first session: *Typically it can be patients who undress themselves from head to toe before I enter the room*.

*Many of our patients coming from non-western cultures are very little process-oriented. They are very impressed by machines and things that flash and beep. And that you manipulate instead of mobilise. And their understanding of pain is very different. I think we are much better at distinguishing pain, [..] and we understand that you have to be active yourself in order to get better which they don't. They expect treatment very explicitly and then the first session is very difficult to set with proper respect (as I need to communicate and examine before treatment)*.

*Many patients from non-western cultures have problems with being questioned by a woman. They kind of do not really respect our competences. They very easily make me feel inferior, and this is clearly an ethical issue. I am the one who should ask the questions and here he comes and take my power.*.

Additionally, patients who did not speak an understandable Danish were found to raise major ethical issues. The interviewees expressed great difficulty with having interpreters granted and involved. In the rare cases where interprets were granted, the first session took a disproportionate amount of time: *It takes ages if you really seek respect and empathy with one person through another person*. Furthermore, the interviewees described a feeling of ongoing uncertainty of mutual understanding due to fundamental different cultural understandings and values expressed in verbal and non-verbal language: *it can be more than difficult to meet this kind of patients with respect because I don't know what they find respectful or violating*.

The male interviewees did not see this as ethical issues and found it to be a practical issue, except for one; *we have decided never to start a session with a patient who does not speak Danish without an interpreter. And we always give female patients to female physios and male patients to male physios. It is very simple. We find this to be respectful and a simple mean to minimise ethical dilemmas that occur about gender and language*.

Some patients do not fit private practice.

Patients who deviated especially from others by being obese, dirty and/or gross, by having severe cognitive damages or by being in the terminal phase, thus calling for ethical reflections, seemed to be difficult for some female interviewees to handle on the personal level in both the first and the following sessions of physiotherapy: *sometimes we have mega-obese patients. I have difficulties meeting them with respect. During the dialogue, I have parallel reflections to the spoken word about how they can stand to eat so much. I find it disgusting! I can't respect them and ask stupid questions. Isn't that an ethical issue?*

The deviation was also difficult for these interviewees to handle on the structural level, due to lack of time and tools to fulfill these patients' fundamental needs; they felt caught between a rock and a hard place, and as one stated: *These patients are not suited for private practice*. The few interviewees who talked about this issue felt that they failed providing initial beneficence towards the patient with the risk to lose the patient's trust in the further process. The rest did not mention this issue.

#### Taking informed consent

The interviewees with post graduate academic education told about taking formal informed consent within the first session or during the entire process of physiotherapy and argued doing so from the perspective of beneficence. They considered informed consent as a process within the physiotherapy-patient relationship which ensured beneficence and patient autonomy and they did not find informed consent to be a formal part of establishing the setting.

When asked, the rest answered in two 'polite' directions; *I haven't got the time*, or *Oh, - oh, of course I take informed consent*. However they could not explain when, how or why.

## Discussion

The study design allowed us to explore the occurrence of ethical issues within the first session and brought forward variations in reflections and actions upon these. The experienced interviewees told of a variety of reflections and actions upon the topic of this study, where others with less experience or the ones with many years of service did not have many reflections, a variation also shown in other studies of ethical issues in physiotherapy [5,13-15,17,18,21,29]. The design showed that the interviewees were able in their discourses to construct reflections upon ethical issues when asked and given time by the repeated interview method. The content of the two interviews did not differ in meaning but the second interview brought forward further refinements and supplements and even new reflections. This strengthens the internal validity and credibility of the results. Credibility and dependability is further strengthened as the study design showed that data convergence is present in the themes but not in every subgroup.

The results showed that the sampling strategy gave a wide range of ethical issues related to the first session. As less experienced practitioners are closer to the basic education we assumed that they would have many theoretical ethical reflections but lack experiences about ethical issues in clinical practice, but as the results show, the less experienced were vague on both aspects. In addition, the results show that interviewees with more academic education expressed an increased ethical awareness in comparison with the rest of the interviewees, which we may interpret in support of the concept of recertification as a possible means to ensure the professionalism of Danish physiotherapists.

### Problems with ethical reflections

The results present a clear picture of the majority of physiotherapists in private practice in shortage and in insecurity of competence when facing ethical issues. Overall, there seems to be an uncertainty about what constitutes an ethical issue, even with experience in daily practice, which earlier research supports [6,13-18,21,29-33]. Ethical issues understood as relational situations where one needs to weigh alternative actions towards a moral problem [8] are mostly described by the experienced interviewees, and especially by the ones with post graduate academic education. They express ethical awareness of their moral actions within the first session and they primarily argue that the value of beneficence is the main ethical aspect.

Some of the issues presented in the results can only be considered ethical in a very broad sense of the concept: the choice of furniture or wall decorations seems more like an aesthetic aspect than an ethical. But when it comes to arguments about having closed rooms for the first session to assure privacy, we too consider it an ethical issue. Without an ethical awareness, the physiotherapist may unintentionally act unethically, where the ethically conscious physiotherapist will know when he or she is acting unethically and therefore be able to alter or adjust the action. Hereby professionalism can be developed in answer to the demands of society [5,7,9,10,12,18,19,21,31,32,34-38], laws [39] and ethical guidelines [40,41].

The majority of the ethical issues related to the first session of physiotherapy can be dealt with simply by focusing on the acquisition of knowledge about how to identify, analyse and solve ethical issues. Trienzenberg recommends that teaching ethics should begin early in the education programme, be integrated throughout the curriculum, and relate to the clinical education. The content should include ethical principles and philosophic theory, awareness of personal ethical values, codes of ethics, and legal issues [15]. Several researchers bring forth suggestions on how to teach ethics [30,32,33,42]. As Edwards et al acknowledge, the knowledge and competence to analyse ethical issues in physiotherapy are as important as the knowledge and competence to analyse biomechanics, both in graduate and postgraduate education [43]. It seems important to stress that ethical knowledge needs to be integrated in all subjects and not just as a self-contained course as ethical issues are embedded in every clinical meeting, reasoning process and practice [5,8,15,31,33,38,42,44]. Nevertheless, Christensen concluded that even when well established ethic education is part of the education curriculum ethical considerations are vague in students' clinical reasoning in clinical practice [45]. Only in 2008 is the actual word "ethics" mentioned in the Danish physiotherapy education curriculum [46] and in chapter 6, §12, the national study programme, it is written that the education in ethics and theory and methods of science is given a total of 3 ECTS.

A common prejudice of private practice is that the attending patients on the whole are well off financially, resourceful and autonomous. Our results present some female physiotherapists who seem to have a low awareness of the interface between personal and professional boundaries which allow personal prejudices about e.g. obesity to shine through their daily practice. If physiotherapists in private practice are not aware of their personal prejudices about age, gender, race, religion, diagnosis, etc, this may have undesirable consequences for the collective of private physiotherapists as these are highly valued by society [2,5,9,10,18,37]. To be professional implies that you always are aware of the professional argumentation of your actions [1,7,8,15,36]. This bring forward the need for supervision of the interface between personal and professional boundaries, the need for education in ethics - but also the need for reconsidering the organisational and structural conditions for different patients entering private practice.

### The importance of the first session

The results stress the importance of the first session as pivotal for establishing a solid setting for the physiotherapy-patient relationship - an importance also found in medical and nursing research [47-49]. The first session relates to moral commitment in physiotherapy private practice: the physiotherapists more or less explicitly express the moral meaning of the first session as actively encouraging the patients to share their suffering. They show moral reflection by understanding the importance of being flexible in order not to invade the patient's personal territory; they reflect on how to arrange the first meeting, they reflect on how their understanding of a trustful relationship relates to consciously trying to establish a respectful and empathic dialogue, where the patient afterwards will describe the physiotherapist as being engaged and actively listening to his suffering. This is in line with the findings of Potter et al [9]. They choose questions that they find respect the integrity of individual patient's and they worry about being respectful when meeting patients from other cultures and traditions. This can be addressed from the perspective of Ethics of Care [8,50] as being the essential ethical understanding of the first meeting. The core notion in an ethics of care understanding is caring for and taking care of others. The theory emphasises traits valued in personal relationships such as e.g. trustworthiness, sympathy and compassion. Particularly the term *caring *refers to care for, emotional commitment to, and deep willingness to act on behalf of persons with whom one has a significant relationship [50] which the results clearly underline.

### Distinguishing between information and communication

The results show that the concept of beneficence is at the core when establishing a good first session. The overall tool for providing beneficence seems to be information. It is well known in physiotherapy that information about the setting, the examination, the diagnosis, the reasoning behind the selected interventions and the prognosis is crucial for effective therapy, and Jensen et al especially stress the importance of teaching patients [51]. However, if the physiotherapist only provides information there a risk of being paternalistic and taking over the patient's lawful right to involvement in her/his own treatment arise [8,39] because information is not the same as communication. It is therefore seems important to distinguish between information and communication. Communication is characterized by mutual respect, engagement, exchange of knowledge and understanding [1,52,53]. By communicating and by giving patients tools to understand themselves we may contribute to mobilising their own resources for self-help [54].

Obtaining a patient's informed consent is a legal requirement [39] and builds on the idea of autonomy, defined as self-governance or self-rule, the individual's capacity for reflection and unforced choice and the freedom to express individual requests and preferences. Obtaining informed consent can therefore be valued as a communicative process [21]. Obtaining informed consent can be applied as a tool for communication rather than just information in physiotherapy private practice. Thereby, the profession can accommodate the stipulated ethical, legal requirements [5,19] and public trust [36]. We suggest that a norm always to obtain oral or written informed consent for physiotherapy interventions with competent patients' call for further interpretation and analysis of what constitutes an informed consent process, especially in the low-risk situations which dominate private practice.

Most of the physiotherapists emphasise, in line with others [52], the need for time to establish a constructive dialogue within the first session. Therefore it is worrying that only few physiotherapists have taken action on this, as all are aware that many ethical issues, implying non-beneficence towards the patient, arise from this specific fact. Making a sound profit within private practice seems to dwarf the ambition of beneficence, which Greenfield earlier has reported [20]. It appears necessary to debate the kind of influence that personal need and greed ought to have on the ethical norm in private practice. Likewise it appears essential to discuss this in relation to an expanding professional autonomy [35]. The physiotherapy profession needs to discuss these issues within the clinics and openly within the profession in order to claim beneficence towards the patient. If society looses faith in physiotherapy the hard gained professional autonomy will be in danger [36,44]. An unambiguous solution to this problem will probably never emerge but an open discussion needs to be initiated.

### The ethical dimension of the clinical context

In accordance with Carpenter et al [19] the results show that the clinical environment influences ethical aspects of the physiotherapy session. Thornquist defines context as the verbal and non-verbal association (the organisational, institutional and material) in which a meeting takes place [1]. The results reveal that the clinical environment primarily is understood as the material decor and only by a few also as the organisational and institutional environment. But as Thornquist stresses that context is continuously changing and it does determine the further interaction [1]. This means that what we say or do in the first session will influence what happens further on and how the involved parties interpret the situation and attach importance to things and events; thereby giving it an ethical dimension. This finding is interesting and calls for further exploration.

## Conclusions

The results showed the first session in private practice to be essential from an ethical perspective. Ethical issues do occur in Danish private practice, consciousness about ethical issues differs, and reflections and actions are based on an inadequate awareness of ethical theories, principles and guidelines. The results stress the need for awareness and self-honesty; consciousness of one's personal resources, values and boundaries as a prerequisite for being professional. The results show that the interviewees reflect on beneficence towards the patient. However, without a deeper ethical awareness, the physiotherapist may reason and/or act ethically to a varying extent: an ethically conscious physiotherapist will be aware of when he or she is reflecting and acting ethically. Further exploration of ethical issues in private practice is needed, and as management policy is deeply embedded within the public sector there are reasons to explore the public contexts of physiotherapy as well.

## Declaration of competing interests

This study has received funding from The Association of Danish Physiotherapists and The Dept. of Physiotherapy, Metropolitan University College, Copenhagen, Denmark and The Danish Rheumatism Association. The authors report no declaration of competing interests.

## Authors' contributions

JP has made substantial contributions to conception and design, acquisition of data, analyses and discussions of data, and has drafted the manuscript. GG has revised the manuscript critically and has given final approval of the version to be published. Both authors have read and approved the final manuscript.

## Pre-publication history

The pre-publication history for this paper can be accessed here:

http://www.biomedcentral.com/1472-6939/12/21/prepub

## References

[B1] Thornquist E Klinik, kommunikation, information 2011 København: Hans Reitzels Forlag

[B2] Poulis I Bioethics and physiotherapy Journal of Medical Ethics 2007 33 435 436 10.1136/jme.2007.021139 PMC2598178 17664296

[B3] Poulis I The end of physiotherapy The Australian Journal of Physiotherapy 2007 53 71 2 10.1016/s0004-9514(07)70038-7 17535141

[B4] Grice RA Ridgeway LS The Evolution of Communication Vehicles: Linear Progress and Cyclical Progress Transactions on Professional Communication 1994 37 3 179 182

[B5] Praestegaard J Etik i fysioterapi Master thesis 2001 Lund Lunds University

[B6] The Association of Danish Physiotherapists The department of member information 2008 http://fysio.dk/praksis/Selvstandig-fysioterapeut/

[B7] Purtillo R Ethical Dimensions In The Health Professions 1999 Philadelphia, Pennsylvania, WB Saunders 12

[B8] Beauchamp TL Childress JF Principles of biomedical ethics 2009 6 New York, Oxford University Press

[B9] Potter M Gordon S Hamer P Identifying physiotherapist and patient expectations in private practice physiotherapy Physiotherapy Canada 2003 55 195 202

[B10] Potter M Gordon S Hamer P The physiotherapy experience in private practice: The patients perspective Australian Journal of Physiotherapy 2003 49 195 202 10.1016/s0004-9514(14)60239-7 12952519

[B11] Potter M Gordan S Hamer P The difficult patient in private practice physiotherapy: A qualitative study Australian Journal of Physiotherapy 2003 49 53 61 10.1016/s0004-9514(14)60188-4 12600254

[B12] Swisher LL A retrospective Analysis of Ethics Knowledge in Physical Therapy (1970-2000) Physical Therapy 2002 82 7 692 706 12088466

[B13] Guccione AA Ethical issues in physical therapy practice: A survey of physical therapists in New England Physical Therapy 1980 60 1264 1272 10.1093/ptj/60.10.1264 7443788

[B14] Barnitt R Patridge C Ethical reasoning in physical therapy and occupational therapy Physiotherapy Research International 1997 4 250 261 10.1002/pri.99 9421822

[B15] Trienzenberg HL The Identification of Ethical Issues in Physical Therapy Practice Physical Therapy 1997 76 10 1097 1107 10.1093/ptj/76.10.1097 8863763

[B16] Barnitt R Truth telling in occupational therapy and physiotherapy British Journal of Occupational Therapy 1994 57 334 340

[B17] Barnitt R Ethical dilemmas in occupational therapy and physical therapy: A survey of practitioners in the UK National Health Service Journal of Medical Ethics 1998 24 193 199 10.1136/jme.24.3.193 PMC1377523 9650115

[B18] Cross S Sim J Confidentiality within physiotherapy: Perceptions and attitudes of clinical practioners Journal of Medical Ethics 2000 26 447 453 10.1136/jme.26.6.447 PMC1733327 11129846

[B19] Carpenter C Richardson B Ethics knowledge in physical therapy: A narrative review of the literature since 2000 Physical Therapy Review 2008 15 5 366 374

[B20] Greenfield BH The meaning of caring in five experienced physical therapists Physiotherapy Theory and Practice 2005 21 147 162 10.1080/09593980600822859 16920677

[B21] Delaney CM Respecting patient autonomy and obtaining their informed consent: Ethical theory - missing in action Physiotherapy 2005 91 197 203

[B22] Dahlberg K Drew N Nyström M Reflective Lifeworld Research 2001 Lund, Studenterlitteratur

[B23] Malterud K The art and science of clinical knowledge: Evidence beyond measures and numbers The Lancet 2001 358 397 400 10.1016/S0140-6736(01)05548-9 11502338

[B24] Malterud K Qualitative Research: Standards, Challenges, and guidelines The Lancet 2001 358 483 88 10.1016/S0140-6736(01)05627-6 11513933

[B25] Malterud K Kvalitative metoder I medisinsk forskning - en innføring 2003 utgave, Oslo, Universitetsforlaget 2

[B26] The Danish Research Ethics Committee http://www.cvk.sum.dk/

[B27] Giorgi A Concerning the application of phenomenology to caring research Scandinavian Journal of Caring Science 2000 14 1 11 15 10.1111/j.1471-6712.2000.tb00555.x 12035256

[B28] Giorgi A (Ed) Phenomenology and Psychological Research 1985 Pittsburgh, USA, Duquesene university Press

[B29] Solomon P Miller PA Qualitative Study of Novice Physical Therapists' Experiences in Private Practice Physiotherapy Canada 2005 57 190 198

[B30] Finch E Geddes EL Larin H Ethically-based clinical decision making in physical therapy: Process and issues Physiotherapy Theory and Practice 2005 21 3 147 162 10.1080/09593980590922271 16389696

[B31] Bruckner J Physical Therapists as Double Agents. Ethical Dilemmas of Divided Loyalties Physical Therapy 1987 67 3 383 386 10.1093/ptj/67.3.383 3823152

[B32] Clawson AL The relationship between clinical decision making and ethical decision making Physiotherapy 1994 80 1 10 14

[B33] MacDonald CA Houghton P Cox PD Bartlett DJ Consensus on Physical Therapy Professional Behaviors Physiotherapy Canada 2001 53 212 222

[B34] May SJ Patient satisfaction with management of back pain. Part 2: An explorative, qualitative study into patient's satisfaction with physiotherapy Physiotherapy 2001 87 10 20

[B35] Rothstein JM Editor's note: Autonomy or professionalism? Physical Therapy 2003 83 206 207 12620085

[B36] Sandstrom RW The meanings of Autonomy for Physical Therapy Physical Therapy 2007 87 98 110) 10.2522/ptj.20050245 17142644

[B37] Bellner A Senses of responsibility: A challenge for occupational and physical therapists in the context of ongoing professionalization Scandinavian Journal of Caring Sciences 1999 13 1 5 10.1080/02839319950162787 10476195

[B38] Magistro CM Clinical Decision Making in Physical Therapy: A Practitioner's Perspective Physical Therapy 1989 69 7 525 534 10.1093/ptj/69.7.525 2740443

[B39] Sundhedsloven LOV nr 319 af 30/04/2008 https://www.retsinformation.dk/Forms/R0710.aspx?id=130455

[B40] World Confederation for Physical Therapy Declaration of Ethical Principles http://www.wcpt.org/node/29031

[B41] The Association of Danish Physiotherapists Ethical guidelines http://fysio.dk/fafo/Etik/Etiske-retningslinjer/

[B42] Purtilo RB Jensen G Royeen C (eds) Educating for Moral Action: A Sourcebook in Health and Rehabilitation Ethics 2005 FA Davis Company

[B43] Edwards I Braunack-Meyer A Jones M Ethical reasoning as a clinical-reasoning strategy in physiotherapy Physiotherapy 2005 91 229 236

[B44] Scott R Supporting professional development: Understanding the interplay between health law and professional ethics Journal of Physical Therapy Education 2000 14 3 17 19

[B45] Christensen N Development of clinical reasoning capability in student physical therapists University of South Australia, PhD thesis 2007

[B46] Bekendtgørelse om uddannelsen til professionsbachelor i fysioterapi https://www.retsinformation.dk/Forms/R0710.aspx?id=120781 BEK nr 831 af 13/08/2008

[B47] Pilnick A Hindmarch J Gill VT Beyond 'doctor and patient': Developments in the study of Healthcare Interactions Sociology of Health and Illness 2009 31 6 787 802 10.1111/j.1467-9566.2009.01194.x 19843267

[B48] Kopelman LM What is Unique About the Doctor and Patient Medical Encounter? A moral and Economic Perspective American Journal of Bioethics 2006 6 2 85 88 10.1080/15265160500507066 16500867

[B49] Sjostedt E Dahlstrand A Severinsson E Lutzen K The first nurse-patient encounter in a psychiatric setting: discovering a moral commitment in nursing Nursing Ethics 2001 8 4 313 27 10.1177/096973300100800404 16004086

[B50] Løgstrup KE Den etiske fordring 1991 København: Nordisk Forlag

[B51] Jensen G Shepard K Gwyer J Hack L Attribute dimensions that distinguish master and novice physical therapy clinicians in orthopedic settings Physical Therapy 1992 72 711 722 10.1093/ptj/72.10.711 1528964

[B52] Westman Kumlin I Krooksmark T The first encounter. Physiotherapist's conception of establishing therapeutic relationships Scandinavian Journal of Caring Science 1992 6 37 44 10.1111/j.1471-6712.1992.tb00121.x 1579770

[B53] Thornquist E Examination and Communication: A Study of First Encounters Between Patients and Physiotherapists Family Practice 1992 9 2 195 2002 10.1093/fampra/9.2.195 1505710

[B54] Gyllensten AL Gard G Salford E Ekdahl C Interaction between patient and physical therapist: A qualitative study reflecting the physical therapist's perspective Physiotherapy Research International 1999 4 2 89 109 10.1002/pri.156 10444760

